# Enhanced methods for unbiased deep sequencing of Lassa and Ebola RNA viruses from clinical and biological samples

**DOI:** 10.1186/s13059-014-0519-7

**Published:** 2014-11-18

**Authors:** Christian B Matranga, Kristian G Andersen, Sarah Winnicki, Michele Busby, Adrianne D Gladden, Ryan Tewhey, Matthew Stremlau, Aaron Berlin, Stephen K Gire, Eleina England, Lina M Moses, Tarjei S Mikkelsen, Ikponmwonsa Odia, Philomena E Ehiane, Onikepe Folarin, Augustine Goba, S Humarr Kahn, Donald S Grant, Anna Honko, Lisa Hensley, Christian Happi, Robert F Garry, Christine M Malboeuf, Bruce W Birren, Andreas Gnirke, Joshua Z Levin, Pardis C Sabeti

**Affiliations:** Broad Institute, 75 Ames Street, Cambridge, MA 02142 USA; FAS Center for Systems Biology, Department of Organismic and Evolutionary Biology, Harvard University, 52 Oxford Street, Cambridge, MA 02138 USA; Tulane Health Sciences Center, Tulane University, 1430 Tulane Avenue, New Orleans, LA 70112 USA; Institute of Lassa Fever Research and Control, Irrua Specialist Teaching Hospital, Irrua, Nigeria; Kenema Government Hospital, Kenema, Eastern Province Sierra Leone; Department of Biological Sciences, College of Natural Sciences, Redeemer’s University, Redemption City, Nigeria; USAMRIID, 1425 Porter, Fort Detrick, Frederick, MD 21702-5011 USA

## Abstract

We have developed a robust RNA sequencing method for generating complete *de novo* assemblies with intra-host variant calls of Lassa and Ebola virus genomes in clinical and biological samples. Our method uses targeted RNase H-based digestion to remove contaminating poly(rA) carrier and ribosomal RNA. This depletion step improves both the quality of data and quantity of informative reads in unbiased total RNA sequencing libraries. We have also developed a hybrid-selection protocol to further enrich the viral content of sequencing libraries. These protocols have enabled rapid deep sequencing of both Lassa and Ebola virus and are broadly applicable to other viral genomics studies.

**Electronic supplementary material:**

The online version of this article (doi:10.1186/s13059-014-0519-7) contains supplementary material, which is available to authorized users.

## Background

Lassa virus (LASV) and Ebola virus (EBOV) belong to a class of RNA viruses that cause hemorrhagic fevers with high case fatality rates, have limited or no treatment options, and have the potential for extensive transmission [1-6]. The need for methods to study these viruses has never been greater. LASV is endemic to many parts of West Africa [1], and EBOV is currently spreading in Guinea, Liberia, Sierra Leone, Senegal, and Nigeria [7]. The current EBOV outbreak has caused approximately 3,000 deaths to date, and is now the largest outbreak, the first in West Africa, and the first to affect urban areas.

LASV and EBOV are both single-stranded RNA viruses. LASV, a member of the *Arenaviridae* family, is an ambisense RNA virus whose genome consists of an L and an S segment of 7.4 kb and 3.4 kb in length, respectively, encoding two proteins on each segment [8]. LASV is transmitted by the multimammate rodent *Mastomys natalensis*, its natural reservoir, which is asymptomatically infected with the virus [9-11]. EBOV belongs to the *Filoviridae* family of single-stranded negative-sense RNA viruses. Its genome is approximately 19 kb in length and it encodes seven proteins [12,13].

LASV and EBOV genomics can inform surveillance, diagnostic, and therapeutic developments, yet few full length genomes have been published [14-16]. The LASV and EBOV whole-genome sequences published prior to our study were sequenced using selective amplification of viral sequences by RT-PCR. Virus-specific primers are however biased towards known strains and variants and do not capture divergent or unknown viruses in the sample.

Massively parallel RNA sequencing (RNA-seq) based on randomly primed cDNA synthesis has the potential to transform LASV and EBOV genomics, providing a comprehensive, largely unbiased qualitative and quantitative view of all RNA in a sample [17-19]. It therefore enables detection and assembly of genomes from highly divergent lineages, unrelated co-infectants, or even novel viruses, making it possible to study viruses that are responsible for fevers of unknown origin and other diseases without known causative infectious agent [20-22]. As a bonus, total RNA-seq can also provide an expression profile of the infected host simultaneously with viral sequence generation.

Sequencing viral genomes directly from clinical and biological samples, however, holds special challenges. Samples may contain very little viral RNA and are heavily contaminated with human RNA; in some instances, the nucleic acid is severely degraded. While poor sample quality affects viral sequencing in general, it is exacerbated for EBOV and LASV. Here, sample quality is often compromised by cold chain gaps in remote rural areas in hot climates and by complications with handling, containment and biological inactivation at the highest biosafety level (US Biosafety Level 4 or equivalent).

The comprehensive and unbiased nature of total RNA-seq also presents a challenge in samples where non-viral RNA makes up the vast majority of material being sequenced. As with most RNA-seq approaches, unwanted RNA contaminants waste many sequencing reads and negatively impact sequencing performance. The largest single component of RNA in clinical samples is human RNA, particularly ribosomal RNA (rRNA). In addition, a prevalent artificial contaminant in RNA preparations is poly(rA) carrier RNA, present in commonly used commercial viral RNA extraction kits (for example, those from QIAGEN and Ambion). Although non-nucleic-acid carriers such as linear polyacrylamide are suitable substitutes, many existing sample collections already contain poly(rA).

Here we describe the development of efficient and cost-effective methods for sequencing of EBOV and LASV that are based on unbiased total RNA-seq. These techniques have already been used to rapidly generate large catalogs of LASV and EBOV genomes ([23], Andersen *et al*., in preparation), including many from the 2014 EBOV outbreak, and can be broadly applied to a wide range of RNA viruses.

## Results

### Challenges of sequencing LASV samples

We initially set out to understand the major issues that arise when sequencing LASV from clinical and biological samples. To do so we prepared 50 RNA-seq libraries directly from human patient and *Mastomys natalensis* samples*.* We performed randomly-primed reverse transcription, followed by second-strand synthesis and ligation of Illumina adapters to the cDNA (see Materials and methods). Two major challenges emerged in our analysis.

**Figure 1:**
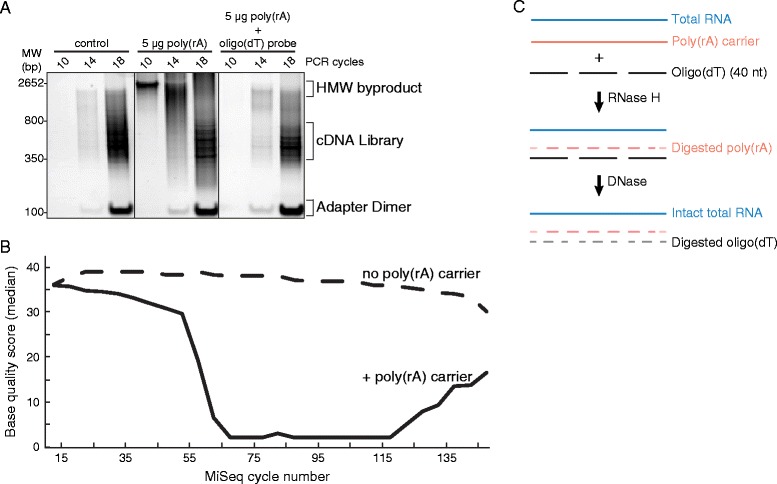
**RNase H selective depletion of poly(rA) carrier from Lassa samples. (A)** Native polyacrylamide gel depicting library PCR and side products of LASV preparations with poly(rA) carrier present (middle) or depleted (right panel). No free poly(rA) was present in control library (left). **(B)** Median base qualities per MiSeq cycle of poly(rA)-contaminated LASV libraries (solid line) and control (no carrier observed in library, dashed) from FastQC report. Both read 1 and read 2 of paired end reads are merged in the library BAM file and the quality scores are shown at each base. **(C)** Schematic of carrier RNA selective depletion and DNase treatment of oligo (dT).

First, we discovered that RNA samples extracted using commercial kits containing poly(rA) RNA carrier resulted in high-molecular-weight byproducts (Additional file 1: Figure S1A). To confirm that these byproducts came from carrier RNA, we added poly(rA) to RNA extracted without carrier and compared the resulting library to a poly(rA)-free control library from the same sample; the high-molecular-weight products were observed only when carrier RNA was added (Figure 1A). Poly(rA) also negatively impacted the raw Illumina sequencing data. As shown in Figure 1B, the median base quality dropped significantly about halfway through the forward and reverse 150-base reads, presumably due to poly(A) reads interfering with calibration of base-calling on the flow cell, while a poly(rA)-free library stayed well above a quality score of 25 until the end of the run.

Second, after sequencing the libraries to >20 million Illumina reads per library, we found that only a small fraction (<0.1%) aligned to the LASV-Josiah reference genome [24] in all but two of the blood isolates (Additional file 1: Figure S1B). A large fraction of reads aligned to the human genome, approximately 75% of them to rRNA. There is also a population of libraries in which host rRNA was low (<40%). In these libraries, a majority of reads did not map to LASV or the host genome. These ‘other’ reads consisted of either low-quality or contaminating reads from bacterial genomes such as *Escherichia coli*, including sequences that were likely introduced during library construction from contaminating nucleic acid in commercial enzyme stocks. For example, reads containing DNA polymerase I sequences aligned exclusively to the coding sequences of the N-terminally truncated Klenow fragment - the enzyme used for the deoxyadenosine addition step during library construction (Additional file 1: Figure S1C). However, ‘other’ reads also aligned to much of the *Escherichia coli* genome, and to many other organisms as well. There was thus no single, obvious source for the contamination (data not shown).

The median fraction of LASV reads in these test libraries was 0.0003% (Additional file 1: Figure S1B), prohibitively low for efficient and cost-effective sequencing at the depth required for *de novo* assembly and for confident calling of intra-host variants. We therefore developed methods to: (1) deplete carrier poly(rA) before library construction; (2) deplete rRNA before library construction; and (3) to enrich LASV reads in libraries before Illumina sequencing. We then demonstrated the utility of these approaches to EBOV sequencing during the 2014 Ebola virus disease (EVD) outbreak.

### Removal of poly(rA) carrier RNA in LASV samples improves sequencing quality

To alleviate the detrimental effects of poly(rA) RNA carrier on sequencing quality, we developed a targeted RNase-H-based depletion method [25] to remove it prior to library construction. We used 40mer oligo(dT) probes to form RNase H-cleavable DNA-RNA hybrids with poly(rA) (Figure 1C), which successfully depleted poly(rA) from a sample with carrier added (Figure 1A; right panel). The depth of sequencing reads along the LASV genome after depletion was similar to the original poly(rA)-free aliquot (Additional file 1: Figure S2), suggesting little off-target hybridization of the oligo(dT) probes.

### Depletion of host rRNA enriches LASV sequences in a variety of samples

**Figure 2:**
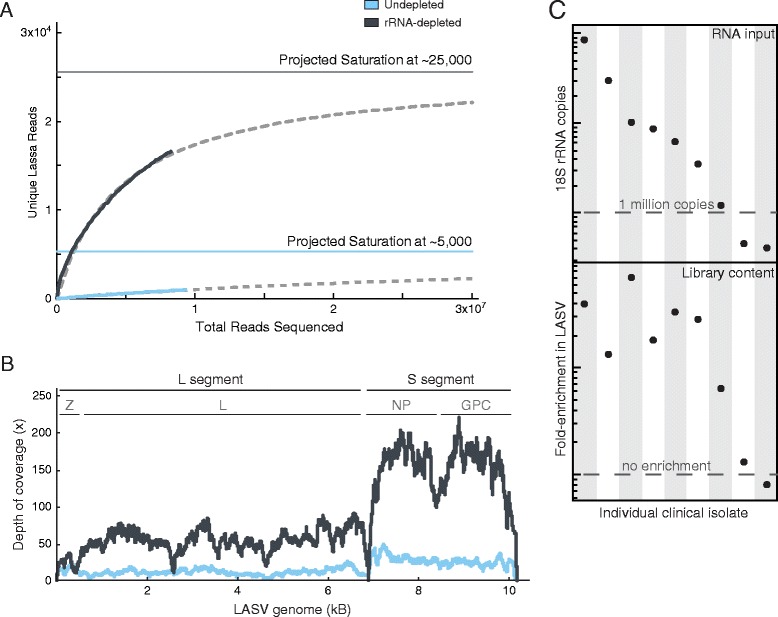
**Depletion of rRNA from human LASV isolates. (A)** Rarefaction analysis of LASV sample (ISTH2016) from a rRNA-depleted (gray) or control (undepleted, blue) preparation. Data best fit (dashed line) to the Michelis-Menten formula in which projected saturation value equals V_*max*_ (see Materials and methods). **(B)** LASV genomic coverage from a LASV sample (ISTH0073) from a rRNA-depleted (gray) or control (blue) preparation. L, S segment, Z, L, NP, GPC: boundaries of each LASV genomic segment with specified genes encoded on each segment. **(C)** Starting overall content (*RNA input*) and enrichment of unique LASV (Library content) upon rRNA depletion from nine different clinical isolates.

To deplete host rRNA in human clinical samples, we pursued selective RNase H-based depletion using oligodeoxyribonucleotides tiled along human cytoplasmic and mitochondrial human rRNA sequences [26]. We achieved almost complete removal of rRNA (from approximately 80% of the reads to less than 1%) with a concomitant enrichment of LASV content in a human plasma sample. As shown by rarefaction analysis of a representative sample (Figure 2A), rRNA depletion increased the unique LASV content in the sequence data to an estimated saturation at approximately 25,000 non-duplicated LASV reads compared to at most 5,000 without depletion.

The host rRNA depletion not only improved overall sequencing depth along the LASV genome (Figure 2B) but revealed finer details of the viral replication dynamics. It uncovered pronounced differences in coverage between the L and S segments, which are known to be present at different copy numbers in infected cells [8]. It also exposed the dip in coverage at the stem-loop between the NP and GPC gene, RNA secondary structure common to many viral genomes [8,27,28].

As most LASV isolates collected from human serum or plasma contain very little total RNA (sub-nanogram levels), we further developed a prescreening process to identify samples suitable for host depletion. We used a real-time qRT-PCR assay for 18S rRNA as a surrogate for quantification of total RNA. We then performed rRNA depletion on nine samples spanning a wide range (approximately 200-fold) of input RNA to determine the minimum amount of RNA required for efficient LASV enrichment. As shown in Figure 2C, our protocol enriched unique LASV content at least five-fold in all samples with at least one million copies of 18S rRNA. Thus, the rRNA selective depletion method can be applied to extremely low-input RNA samples containing as little as picograms of total RNA. In comparison to previous selective RNase H depletion publications [25,26], our method was successful with approximately 1,000-fold less material.

**Figure 3:**
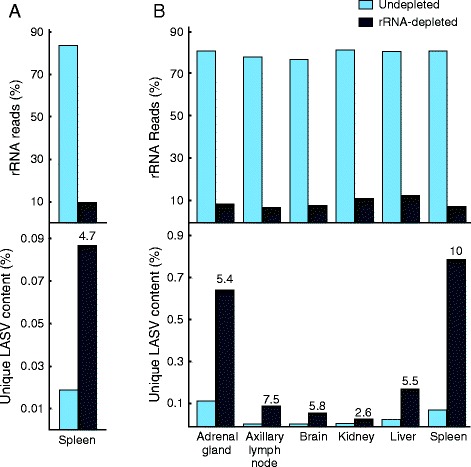
**Depletion of rRNA from rodent and macaque LASV isolates. (A)** Depletion of rRNA (top) and unique LASV (bottom) enrichment from *Mastomys natalensis* spleen and **(B)** various tissues from cynomolgous macaque (day 12 post LASV infection). Numbers over fraction unique reads represent fold-enrichment in LASV content after rRNA depletion.

We demonstrated the utility of host rRNA depletion on tissue samples collected from LASV-infected rodents and non-human primate disease models. These tissue samples contain higher levels of 18S rRNA than human plasma or serum (on average 5 times more - data not shown). Using the same human rRNA probes, we depleted rRNA and enriched unique LASV reads approximately five-fold in a *Mastomys natalensis* spleen sample (Figure 3A). Most of the remaining 10% (approximately) rRNA reads aligned to 28S rRNA sequences which are divergent between humans and rodents [29]. Similarly, our protocol reduced the rRNA content in six different tissue samples from cynomolgous macaques to approximately 10% (Figure 3B). Depletion of rRNA led to an increase in LASV content in all macaque samples, reaching the highest levels in adrenal gland and spleen, two tissues known to accumulate LASV during infection [30].

### Hybrid selection of sequencing libraries rescues LASV genomes

Despite efficient depletion of carrier RNA and host rRNA, in a number of cases the fraction of LASV sequencing reads stayed well below 1%. For these samples, sequencing to the depth required for *de novo* assembly of LASV genome (>10×) and for detecting intra-host variants with minor allele frequencies as low as 5% (>100×) remains cost prohibitive.

In order to capture LASV genomes in ultra-low coverage libraries, we used solution hybrid selection [31,32] to further enrich the LASV content of sequencing libraries. Hybrid selection has been previously shown to effectively capture pathogen sequence in difficult clinical samples [33]. We designed a complex set of 42,000 100mer oligonucleotides based on a diverse set of consensus LASV genomes sequenced using our host rRNA depletion protocols (Andersen *et al.*, in preparation). We then synthesized the oligonucleotides on a microarray, PCR-amplified them as a pool, and prepared single-strand biotinylated RNA baits for hybrid capture [31].

We tested the LASV hybrid selection method on a set of 13 libraries from different sample sources (human, *Mastomys*) and geographical regions (Nigeria, Sierra Leone) that had been previously sequenced (Andersen *et al*., in preparation). This test set included libraries that contained high host content (that is, rRNA and mRNA) or produced poor LASV genome coverage. We also included libraries with low duplication rates indicating under-sampling of LASV sequences. These libraries may potentially contain unique LASV sequences that were masked by host or other contaminating content in the library.

The average enrichment of unique LASV content in the sequencing data was 86-fold (Additional file 1: Table S1; median enrichment, 9.6-fold; range, approximately 2 to 724). We note that the hybrid-selected libraries were sequenced to a higher degree of saturation with generally much higher duplication rates including four data sets with >99% duplicate reads (samples G2230, ISTH0230, ISTH1137, LM032). Nonetheless, the average coverage of the LASV genome with unique, non-duplicate reads reached approximately 1,080× (Table 1 and Additional file 1: Table S2; range, 5 to 1,083×; median (average) coverage, 53×). We performed rarefaction analysis of libraries from a representative sample (Additional file 1: Figure S3; ISTH1137) to illustrate the greater LASV sequence complexity in hybrid selection libraries compared to standard libraries at lower read depths (max sampling, 4 million reads).

**Table 1:** **LASV genome coverage from standard RNA-seq and hybrid selection libraries**

	**Standard**				**Hybrid selection**			
**LASV sample**	**Total reads (×10** ^**6**^ **)**	**Median coverage**	**Normalized coverage** ^**a**^	**Assembled LASV genome?**	**Total reads (×10** ^**6**^ **)**	**Median coverage**	**Normalized coverage** ^**a**^	**Assembled LASV genome?**
G090	5.2	1	0.28	No	1.2	20	19.25	Yes
G2230	1.3	2	7.73	No	1.2	1	24.84	No
G733	6.9	85	17.18	Yes	1.3	527	636.71	Yes
G771	24.5	65	3.55	Yes	2.5	14	12.56	Yes
ISTH0073	35.0	115	3.86	Yes	1.5	208	197.28	Yes
ISTH0230	7.3	4	0.33	No	1.3	6	4.28	Yes
ISTH1137	8.1	18	2.86	Yes	8.0	47	6.84	Yes
ISTH2020	8.9	28	5.26	Yes	1.2	53	78.84	Yes
ISTH2025	40.2	13	0.60	Yes	1.2	30	43.83	Yes
ISTH2050	6.9	20	3.44	Yes	1.2	18	41.94	Yes
LM032	14.9	121	8.99	Yes	12.3	1,003	88.18	Yes
LM222	6.3	6	0.96	Yes	2.6	390	158.73	Yes
Z002	5.8	0	0.08	No	1.1	23	26.09	Yes

^a^Average base coverage per 1 million reads. Successful LASV genome assembly required >1× coverage of 90% of LASV ORF covered. Coverage metrics are based upon unique, non-duplicated LASV reads. G-series: Sierra Leone clinical isolates (4). ISTH series: Nigeria clinical isolates (6). LM and Z series: *Mastomys natalensis* isolates. Other metrics including average (×) coverage and % genome coverage at >1× are included in Additional file 1: Table S2.

**Figure 4:**
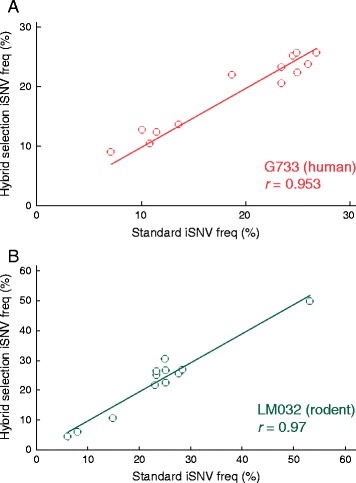
**Hybrid selection of LASV.** Frequencies of intra-host variants (iSNVs) observed in **(A)** human (*G733*) and **(B)** rodent (*LM032*) in standard and hybrid selected libraries. Data fit to a linear regression with y-axis intercepts set at 0. *r*: Pearson correlation value.

The hybrid selection approach not only lowers the cost of sequencing, but is a powerful approach for characterizing viral genomes. Only two of the original libraries provided enough coverage to call intra-host single nucleotide variants (iSNVs) at high confidence (13 and 12, respectively). In both cases, hybrid selection increased the number of detectable iSNVs (to 21 and 29, respectively). Importantly, none of the 25 previously observed iSNVs dropped out during the selection process (Additional file 1: Tables S3 and S4). Furthermore, the correlation of the allele frequencies before and after hybrid selection was excellent (r = 0.95 and 0.97; Figure 4A and B), indicating that hybrid selection with our LASV bait introduces little, if any, allelic bias. This is consistent with data reported for human exome sequencing [31]. Moreover, four of the initial 13 libraries failed to produce complete *de novo* assemblies of the LASV genome, despite approximately 5 to 7 million reads generated per library. In contrast, after hybrid selection, three of these four samples yielded complete *de novo* assemblies from only slightly more than one million reads each (Table 1).

### rRNA depletion and deep sequencing of EBOV genomes from the 2014 outbreak

As we were completing our study of LASV, we were asked to take on a new effort to sequence EBOV clinical samples when the 2014 outbreak spread to our research site in Sierra Leone. As our poly(rA) and host rRNA depletion approach had worked well with a wide range of clinical LASV samples we examined its utility on the first cases from the outbreak in Sierra Leone [16]. We sequenced four individual clinical isolates with and without poly (rA) and rRNA depletion and generated approximately one million Illumina reads per library.

**Figure 5:**
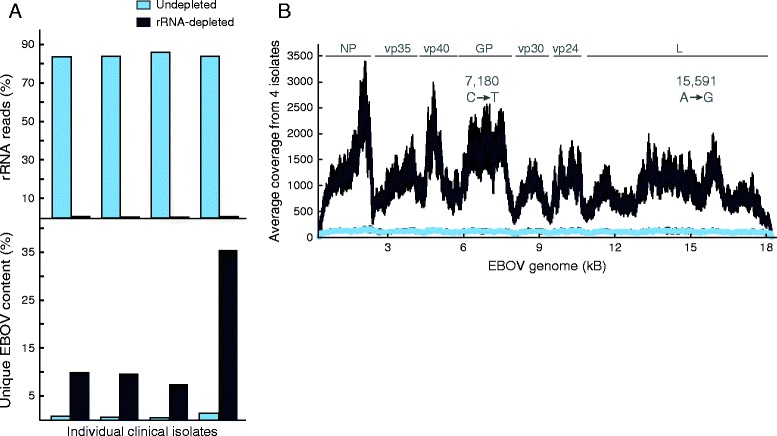
**Depletion of rRNA from EBOV-Sierra Leone clinical samples. (A)** Percentage rRNA (left) and unique EBOV content (right) with (gray) and without (blue) rRNA depletion in four individual clinical serum isolates (G3676-2, G3677-1, G3677-2, G3682-1). **(B)** Average EBOV genome coverage with (gray) and without (blue) rRNA depletion from four individual isolates with standard deviation (black). N, VP35, VP40, GP, VP30, VP24, L: boundary for each gene in the EBOV genome. Positions and variant allele of two iSNVs (in G3676-2 only) observed after rRNA depletion are depicted.

Using our approach, we were able to lower the rRNA contamination in all four samples from >80% to <0.5% (Figure 5A). The concomitant increase of EBOV content was approximately 13- to 24-fold, with unique content reaching approximately 35% of total reads in one of the rRNA depleted libraries. Although we sequenced eight libraries on a single MiSeq run, we achieved >50× average coverage for 99% of the EBOV genome (Figure 5B).

The host rRNA depletion similarly enabled better characterization of the viral genome. We called two iSNVs with >5% minor allele frequency in a single sample (approximate position indicated in Figure 5B); these iSNVs did not reach the detection threshold in the undepleted sample. The pattern of coverage along the EBOV genome was very consistent across all samples, with pronounced dips largely corresponding to boundaries between genes. Coverage levels likely mirror the expression levels of individual genes during EBOV replication [13]. As with LASV, these details could only be resolved with higher coverage of EBOV seq made possible by efficient depletion of rRNA (Figure 5B).

## Discussion

We have overcome key technical challenges in deep RNA sequencing and *de novo* assembly of LASV and EBOV genomes. We have shown that both poly(rA) and rRNA contaminants can be efficiently removed by targeted RNase H-based digestion prior to library construction. Selective depletion is a cost-effective, high throughput alternative to size-selection for removing unwanted carrier RNA from viral samples. Since we are selectively depleting rRNA in our current protocol, there are no added steps when depleting carrier RNA. Further, depletion of poly(rA) prior to cDNA synthesis limits homopolymer A and T sequence in final libraries, resulting in cleaner preparations and ensuring higher quality sequencing runs.

Enrichment by rRNA depletion allowed unbiased total RNA-seq while still achieving sufficient coverage for *de novo* genome assembly and detection of iSNVs in approximately two-thirds of our LASV samples. Moreover, the increased coverage permits deeper exploration of the genome: systematic unevenness along the genome, while it may in part be due to experimental biases, suggests biological features in genome organization such as stem-loop structures between genes and differences in segment copy numbers and expression levels during replication (Figures 2 and 5). Strand-specific RNA-seq methods [26] may help discriminate between the viral genome and complementary RNA intermediates within the viral population.

We were able to enrich for viral content in two distinct RNA viruses and in a variety of sample types, often with very low input of RNA. EBOV and LASV are quite different ssRNA viruses - one negative-sense and one segmented - and our method significantly increases the viral content in sequencing libraries from both. The approach worked well with samples that included human blood from clinical sources (Figures 2 and 5), and rodent and non-human primate tissues (Figure 3). Depletion of rRNA effectively enriched viral RNA in samples containing as few as one million rRNA molecules. For ultra-low-input samples, cDNA-amplification methods such as Ovation RNA-seq (NuGEN) may be more suitable [34], although interference by poly(rA) carrier in the input RNA would need to be overcome for samples including it.

Our approach, while designed for LASV, enables robust, universal, rapid sequencing and was readily transferrable to sequencing EBOV during the 2014 outbreak. We had initially developed and implemented our techniques to generate over 300 LASV genomes from Nigeria and Sierra Leone, and from humans and *Mastomys*. When an outbreak spread to our field site in Sierra Leone, we were able to quickly apply our technology to sequence 99 EBOV genomes from 78 patients in Sierra Leone to approximately 2,000× coverage, processing two batches of samples each within 1 week. By successfully pairing our approach with Nextera (Illumina) library construction, we are able to decrease the overall process time three-fold. We were thus rapidly able to make our data available to the community, to enable timely insights for surveillance and control efforts and to inform diagnostic and therapeutic developments during the epidemic.

Hybrid selection in RNA-seq libraries can further enrich for virus in ultra-low input samples and can also serve as a cost-effective first-line sequencing method. As our data and previous exome studies indicate that single-base mismatches between target and bait sequences cause little allelic bias (Figure 4), future bait designs may containing fewer variants but instead targeting more viruses. This multi-virus hybrid selection could rescue unbiased total-RNA-seq libraries that did not yield complete assemblies and could indeed itself become a first-line sequencing method. The more expensive total-RNA-seq could be reserved for those samples that are not captured by the hybrid selection array. This approach may prove efficient for examining a variety of sample types (serum, nasal aspirate, spinal tap, urine, and so on) and enable many labs around the world to more rapidly detect a wide variety of viruses causing disease in their home countries.

## Conclusion

Our newly developed viral sequencing protocol combines selective depletion of contaminating carrier RNA and host rRNA with unbiased total RNA-seq of randomly-primed cDNA. It thereby improves the quality of raw sequencing data and boosts the fraction of unique informative reads, producing sufficient LASV and EBOV reads for *de novo* genome assembly and intra-host variant calls in diverse clinical and biological samples. Our RNase H-depletion-RNA-seq method may be more broadly applicable to sequence and assemble the genomes of many RNA viruses, known or unknown. We also developed a hybrid selection method to enrich viral content of libraries prior to sequencing, significantly lowering the cost of sequencing and rescuing RNA-seq libraries with very low coverage. While enrichment by hybrid selection requires prior sequence knowledge, hybrid selection with a complex multi-virus bait may prove to be a broadly applicable, viable and cost-effective approach to sequencing.

## Materials and methods

### Ethics statement

Lassa fever patients were recruited for this study using protocols approved by human subjects committees at Tulane University, Harvard University, Broad Institute, Irrua Specialist Teaching Hospital (ISTH), Kenema Government Hospital (KGH), Oyo State Ministry of Health, Ibadan, Nigeria, and Sierra Leone Ministry of Health. All patients were treated with a similar standard of care and were offered the drug Ribavirin, whether or not they decided to participate in the study. For Lassa fever (LF) patients, treatment with Ribavirin followed the currently recommended guidelines [9] and was generally offered as soon as LF was strongly suspected.

Due to the severe outbreak for Ebola Virus Disease (EVD), patients could not be consented through our standard protocols. Instead use of clinical excess samples from EVD patients was evaluated and approved by Institutional Review Boards in Sierra Leone and at Harvard University. The Office of the Sierra Leone Ethics and Scientific Review Committee, the Sierra Leone Ministry of Health and Sanitation, and the Harvard Committee on the Use of Human Subjects have granted a waiver of consent to sequence and make publically available viral sequences obtained from patient and contact samples collected during the Ebola outbreak in Sierra Leone. These bodies also granted use of clinical and epidemiological data for de-identified samples collected from all suspected EVD patients receiving care during the outbreak response. The Sierra Leone Ministry of Health and Sanitation also approved shipments of non-infectious non-biological samples from Sierra Leone to the Broad Institute and Harvard University for genomic studies of outbreak samples.

### Sample collections and study subjects

Human samples were obtained from patients with LF; all samples were acquired on the day of admission before any treatment regimens had been started. The time from onset of symptoms to admission at the hospital was similar between patients from Sierra Leone and Nigeria (average values, Sierra Leone = 9.3 days (range, 0 to 20 days); Nigeria = 9.7 days (range, 0 - 30 days)). Human samples were obtained from patients suspected with EVD and stored in -20°C freezers; samples were collected using existing collection and processing protocols at Kenema Government Hospital (KGH), under the emergency response efforts established by KGH. For LF and EVD samples, 10 mL of whole blood was collected and plasma or serum was prepared by centrifugation at 2,500 rpm for 15 min. Diagnostic tests for the presence of LASV were performed on-site using PCR [35] and/or ELISA antigen capture assays [36]. Both assays have comparable sensitivity [37]. Diagnostic tests for the presence of EBOV were performed using on-site PCR [38]. All samples were re-tested by PCR upon receipt at Harvard University.

Rodents (all from Sierra Leone) were trapped in case-households, humanely sacrificed, and samples were collected from spleens.

Previously collected cynomolgous macaques tissue samples were used [39] from macaques exposed via aerosol to a target dose of 1,000 PFU of LASV Josiah at the United States Army Medical Research Institute of Infectious Diseases (USAMRIID) biosafety level 4 laboratory. Aerosols were created by an automated bio-aerosol exposure system using a 3-jet Collison nebulizer (BGI, Inc., Waltham, MA, USA). Samples were used from day 12 post infection.

All viral samples were inactivated in AVL buffer (Qiagen) or TRIzol (Life Technologies) following standard operating procedures. Samples were stored in liquid nitrogen or at -20°C. In some cases, RNA was isolated at the clinical site using the QIAamp Viral RNA Minikit (Qiagen), lyophilized using RNAstable (Biomatrica) (all according to the manufacturer’s protocol) and stored at room temperature in desiccator cabinets. Inactivated samples were shipped on dry ice to Tulane or Harvard University and stored at -80°C (all samples) or room temperature (Biometrica) until further processing.

### Viral RNA isolation

RNA (from AVL) was isolated using the QIAamp Viral RNA Minikit (Qiagen) according to the manufacturer’s protocol, except that 0.1 M final concentration of β-mercaptoethanol was added to each sample. RNA (from Trizol) was isolated according to the manufacturer’s protocol with slight modifications. Briefly, 200 μL 1-bromo-2 chloropropane (BCP) was added for every 1 mL TRIzol used. After phase separation, 20 μg of linear acrylamide was added to the aqueous phase. All extracted RNA was resuspended in water and treated with Turbo DNase (Ambion) to digest contaminating DNA.

### Quantification of RNA content using qRT-PCR

Host RNA (18S rRNA) were quantified using the Power SYBR Green RNA-to-Ct 1-Step qRT-PCR assay (Life Technologies) and human 18S rRNA primers (5′-CCTGAGAAACGGCTACCACATC-3′ (forward), 5′-AGAGTCCTGTATTGTTATTTTTCGTCACT-3′ (reverse)). Human genomic DNA (Promega) was used as a standard control. All reactions were performed on the ABI 7900HT (Applied Biosystems).

### Carrier RNA and host rRNA depletion

Poly(rA) and host rRNA was depleted using RNase H selective depletion [26]. Briefly, 616 ng oligo (dT) (40 nt long) and/or 1,000 ng DNA probes complementary to human rRNA were hybridized to 5 μL sample RNA in 10 μL. The sample was then treated with 20 units of Hybridase Thermostable RNase H (Epicentre) for 30 min at 45°C. The complementary DNA probes were removed by bringing the reaction up to 75 μL and treating with RNase-free DNase kit (Qiagen) according to the manufacturer’s protocol. rRNA-depleted samples were purified using 2.2× volumes AMPure RNA clean beads (Beckman Coulter Genomics) and eluted into 10 μL water for cDNA synthesis.

### Illumina library construction and sequencing

For the experiments in this study, selectively-depleted EBOV and LASV RNA were fragmented for 4 minutes at 85° C using NEBNext Fragmentation buffer (New England Biolabs). After fragmentation, samples were purified using 2.2x volume AMPure RNA clean beads (Beckman Coulter Genomics). In the production protocol implemented after this study we removed the fragmentation step [23]. Random-primed cDNA synthesis and Illumina paired-end library construction followed the previously published RNase H libraries protocol [26] with some modifications. First, controls were used to monitor our library construction process. We spiked in 1 pg of one, unique synthetic RNA (ERCC, [40] using a different RNA for each individual sample to aid in tracking our viral sequencing process and potential index cross-contamination. Libraries were prepared from human K-562 total RNA (Ambion) with each batch as a control. Second, we removed poly(rA) carrier, high molecular weight products. For some of the initial library preps and for method comparison, we removed longer products using a time-course Pippen Prep (Sage Science) to collect all material <2 kb. In our current protocol, we use the selective depletion approach to remove carrier RNA (see above). Third, we generally used six to 18 cycles of PCR to generate our libraries from 10% to 40% of the adapter-ligated product. Each individual sample was indexed with an 8 bp unique barcode and libraries were pooled equally and sequenced on the HiSeq2000 (101 bp paired-end reads; Illumina), the HiSeq2500 (101 or 150 bp paired-end reads; Illumina), or the MiSeq (150 bp paired-end reads; Illumina) platforms.

### Hybrid selection

Bait design and hybrid selection was done similarly to a previously published method [31]. Briefly, baits were designed by first concatenating all LASV consensus sequences into two single bait sets (one for Nigerian clades and another for the Sierra Leone clade, see Additional file 2). Duplicate probes, defined as a DNA sequence with 0 mismatches, were removed. The baits sequences were tiled across the LASV genome creating a probe every 50 bases. Two sets of adapters were used for each bait set. Adapters alternated with each 50 base probe to allow separate PCR amplification of two non-overlapping sets of oligos for each bait set. The oligo array was synthesized on a CustomArray B3 Synthesizer, as recommended by the manufacturer, and amplified by two separate PCR reactions with primers containing T7 RNA polymerase promoters. Biotinylated baits were then prepared through *in vitro* transcription (MEGAshortscript, Ambion). RNA baits for each clade were prepared separately and mixed at the equal RNA concentration prior to hybridization. LASV libraries were added to the baits and hybridized over a 72 h. After capture and washing, libraries were amplified by PCR using the Illumina adapter sequences. Libraries were then pooled and sequenced on the MiSeq platform.

### Demultiplexing of sequencing runs and QC

Raw sequencing reads were demultiplexed using the Picard v1.4 pipeline [41] and saved as BAM files [42]. To avoid barcode cross-contamination between samples the default settings were changed to allow for no mismatches in the barcode and a minimum quality score of Q25 in the individual bases of the index. Sequencing quality metrics were collected using FastQC v0.10.0 [43] and only high-quality sequencing libraries were used in subsequent analyses.

### Assembly of full-length LASV and EBOV genomes

BAM files were converted to Fastq format and then all viral reads were extracted prior to *de novo* assembly. This was done using the program Lastal r247 [44] with a custom-made database containing full-length filovirus (EBOV) or arenavirus (LASV) genomes. Since the reads are not strand specific our assemblies and iSNV calls (see below) represent the viral genome, the cRNA and mRNAs. All viral Lastal-aligned readswere *de novo* assembled using Trinity r2011-11-26 with a minimum contig size of 300 [45]. Contigs were oriented and manually curated in the software package Geneious v6.1. Once contigs had been generated, all sequencing reads from individual samples were aligned back to its own EBOV and LASV consensus using Novoalign v2.08.02 (Novocraft) with the following stringent parameters -k -l 40 -g 40 - × 20 -t 100. Duplicates were removed using Picard v1.4 and BAM files were locally realigned using GATK v2.1 [46]. If multiple sequencing runs had been performed for the same sample, BAM files were merged using Picard v1.4 before further analyses. Consensus sequences were called using GATK v2.1. All generated genomes were manually inspected, checked, and corrected for accuracy, such as the presence of intact ORFs, using Geneious v6.1. Regions were depth of coverage was less <2× were called as ‘N’. Samples that failed to generate high-quality consensus sequences were excluded from all further analyses.

### Alignment to viral, host, and bacterial reference genomes

To determine the composition of each library, reads were aligned to viral and host references as previously described [34]. The reference genomes used were human genome assembly (GRCh37/hg19), human rRNA sequences (NR_003286.1, NR_003287.1, V00589.1, NR_003285.2, gi|251831106:648-1601, gi|251831106:1671-3229), and viral reference (LASV or EBOV consensus; submissions in process). To identify the bacterial contaminants, reads were aligned to the *E.coli* full genome (gi|48994873) or DNA polymerase I (polA, NC_000913.3).

### Rarefaction analysis

Rarefaction analysis was performed by down sampling the reads at 200 intervals using using custom scripts [47,48]. For each sampling, we counted the number of unique reads. Reads where both fragments of the read aligned at the same starting position were considered PCR duplicates of the same molecule and were counted as a single unique read. Saturation points were estimated by fitting the data to the Michealis-Menten equation using curve fitting tool (MATLAB) (Figure 2A).

### Intra-host variant calling

Reads were realigned to a consensus sequence and variants were called using mpileup: samtools mpileup -Q 0 -B -q 1 -d 10000 and VarScan v2.3 [49] with the following parameters: varscan.jar pileup2snp --min-reads2 5 --min-var-freq 0.01 --p-value 0.1 --min-coverage 5 --min-avg-qual 5. Stringent post-call filtering variables were applied including minimums of overall coverage (5×), frequency (5%), and base quality (q25).

### Data availability

Next-generation viral RNA-seq data can be found in the NCBI database [50] under Bioproject numbers PRJNA254017 (LASV) and PRJNA257197 (EBOV). See Additional file 3 for accession numbers.

## Additional files

Additional file 1: **Supplementary material including: Figures S1 to S3, Tables S1 to S3.**

Additional file 2: **Probe design for Lassa virus hybrid selection.**

Additional file 3: **Accession numbers for data submission.**

## Abbreviations

EBOV: Ebola virus
EVD: Ebola virus disease
iSNVs: intra-host single nucleotide variants
LASV: Lassa virus
LF: Lassa fever
poly(rA): polyriboadenosine
qRT-PCR: quantitative reverse transcription-polymerase chain reaction
rRNA: ribosomal RNA
