# Supplementary material for: Enhanced methods for unbiased deep sequencing of Lassa and Ebola RNA viruses from clinical and biological samples
Source: Genome Biol. 2014 Nov 18;15(11):519. doi: 10.1186/s13059-014-0519-7 (PMC4262991; doi:10.1186/s13059-014-0519-7)
Supplement: Supplementary file 1 — Supplementary material including: Figures S1 to S3, Tables S1 to S3. [file 13059_2014_519_MOESM1_ESM.pdf]

Supplementary material: Matranga et al., ***Enhanced methods for unbiased deep sequencing of Lassa and Ebola RNA viruses in clinical and biological samples***

Contents:

Supplementary Figures 1 to 3

Supplementary Tables 1 to 3

Supplementary Figure 1. Challenges of sequencing LASV samples. (A) We discovered high molecular weight products (>2 kB) in LASV samples extracted with poly(rA) carrier. *****: Library concatemers from PCR overcycling. **(B)** Box-plot depicting fraction of each library (n=50) aligned to LASV (Josiah reference, median fraction, 0.0003), to human (hg19 genome, median fraction, 0.256), or remaining fraction ('other', median fraction, 0.70). Libraries were prepared from LASV samples that were selected randomly with only prior knowledge of a LASV "positive" diagostic in the clinic (human) or trapping (rodent) site. Quantiles represent median, lower 25 and upper 75 percentil**e (C).** Reads from three LASV libraries (*G1180, G610, G1932*) were aligned to E.coli DNA polymerase 1 gene, polA. Boundaries for N-terminal exonuclease and C-terminal polymerase (*Klenow fragment*) depicted.

Supplementary Figure 2. LASV genome coverage after carrier depletion protocol. Two libraries prepared from LASV sample ISTH0073 with poly(rA) carrier RNA in which poly(rA) was removed before (*carrier RNA depletion*, grey) or after library prep (*Pippen prep*, blue) (Sage Science), were sequenced and resulting reads were aligned to reference genome.

Supplementary Figure 3. Rarefaction analysis of hybrid selection. Rarefaction analysis of libraries (from ISTH1137) before (*no selection*) and after (*hybrid selection*) hybrid capture.

Supplementary Figure 1

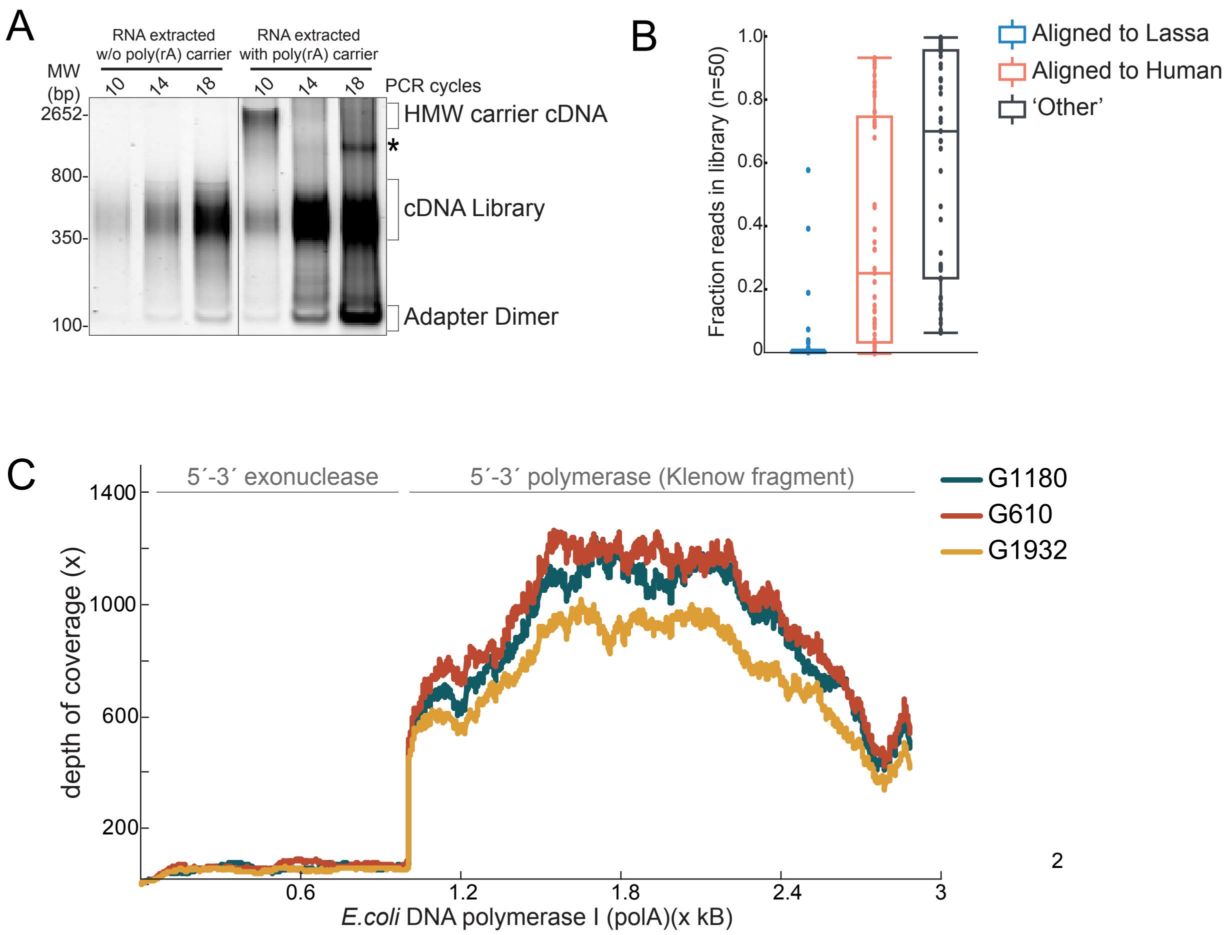

Supplementary Figure 2

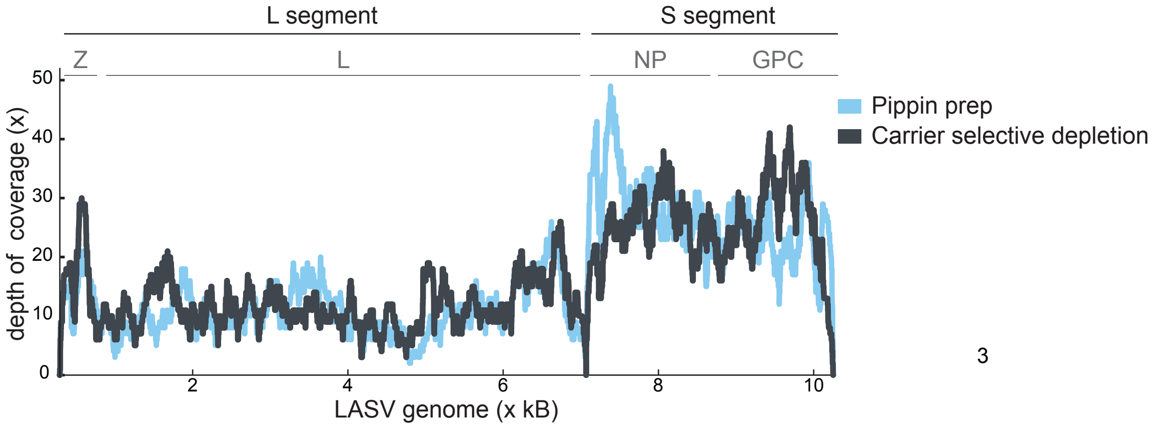

Supplementary Figure 3

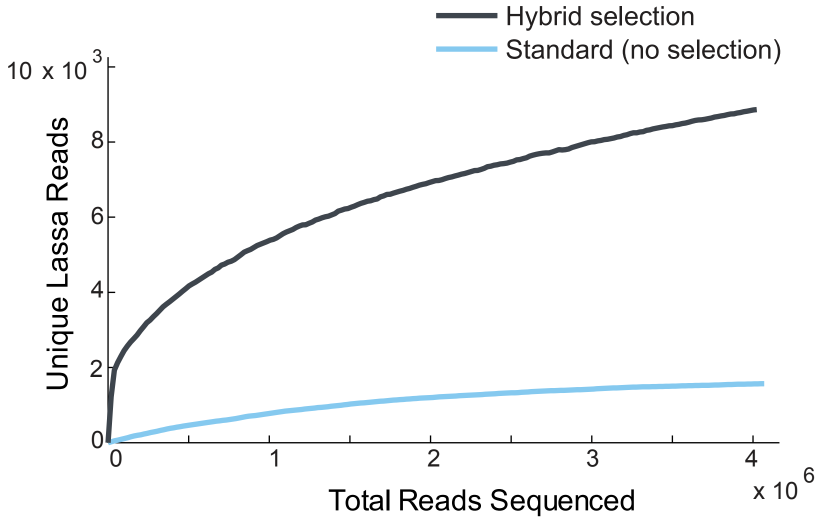

Supplementary Table 1. LASV sequence enrichment after hybrid selection.

LASV sample	Fold enrichment[A]
G090	43
G2230	3
G733	34
G771	3
ISTH0073	52
ISTH0230	8
ISTH1137	2
ISTH2020	8
ISTH2025	66
ISTH2050	6
LM032	10
LM222	153
Z002	724

[A]Fold difference in average base coverage between standard library (downsampled to hybrid selection read depth) and hybrid selection library. Enrichment metrics are based upon unique, non-duplicated LASV reads. G-series: Sierra Leone clinical isolates (4). ISTH series: Nigeria clinical isolates (6). LM and Z series: *Mastomys natalensis* isolates.

Supplementary Table 2. LASV genome coverage after hybrid selection.

	Standard						Hybrid Selection					
LASV sample	Total reads ($x10^6$)	Average coverage	Median coverage	Normalized coverage[A]	>1x coverage (% genome)	Assembled LASV genome?	Total reads ($x10^6$)	Average coverage	Median coverage	Normalized coverage[A]	>1x coverage (% genome)	Assembled LASV genome?
G090	5.2	1	1	0.28	40.36	No	1.2	23	20	19.25	99.39	Yes
G2230	1.3	10	2	7.73	59.51	No	1.2	31	1	24.84	47.68	No
G733	6.9	119	85	17.18	99.67	Yes	1.3	833	527	636.71	99.09	Yes
G771	24.5	87	65	3.55	99.83	Yes	2.5	32	14	12.56	99.85	Yes
ISTH0073	35.0	135	115	3.86	99.95	Yes	1.5	289	208	197.28	99.95	Yes
ISTH0230	7.3	2	4	0.33	79.91	No	1.3	5	6	4.28	89.36	Yes
ISTH1137	8.1	23	18	2.86	99.93	Yes	8.0	55	47	6.84	99.93	Yes
ISTH2020	8.9	47	28	5.26	99.95	Yes	1.2	96	53	78.84	99.93	Yes
ISTH2025	40.2	24	13	0.60	99.91	Yes	1.2	53	30	43.83	99.96	Yes
ISTH2050	6.9	24	20	3.44	99.96	Yes	1.2	49	18	41.94	97.26	Yes
LM032	14.9	134	121	8.99	99.93	Yes	12.3	1,082	1,003	88.18	99.97	Yes
LM222	6.3	6	6	0.96	94.53	Yes	2.6	418	390	158.73	99.61	Yes
Z002	5.8	0	0	0.08	11.95	No	1.1	29	23	26.09	99.45	Yes

[A]Average base coverage per 1 million reads. Successful LASV genome assembly required >1x coverage of 90% of LASV ORF covered. Coverage metrics are based upon unique, non-duplicated LASV reads. G-series: Sierra Leone clinical isolates (4). ISTH series: Nigeria clinical isolates (6). LM and Z series: *Mastomys natalensis* isolates.

Supplementary Table 3. iSNV calls in LASV G733 human libraries

			**Standard (n=13)**		**Hybrid selection (n=21)**	
LASV segment	LASV genome position	Reference allele	Variant allele	Variant frequency (%)	Variant allele	Variant frequency (%)
L	1,303	T	G	25.0	G	22.3
L	2,821	A	G	23.4	G	23.2
L	3,868	C	T	23.5	T	20.5
L	4,331	A	G	18.7	G	21.9
S	286	T	C	26.0	C	23.7
S	713	A	G	10.1	G	12.7
S	1,303	G	A	24.6	A	25.1
S	1,851	G	A	24.9	A	25.7
S	1,962	A	G	26.8	G	25.7
S	2,248	T	C	10.9	C	10.4
S	2,727	C	T	11.5	T	12.3
S	2,754	C	T	7.1	T	9.0
L	272	T			C	11.0
L	379	A			G	11.5
L	656	C			T	8.1
L	1600	G			A	9.9
L	5209	T			C	9.5
L	6208	C			T	12.1
L	6427	T			C	12.4
S	199	T			C	10.3

All variants for standard and hybrid selection (> 5 minor allele frequency) called using VarScan (see methods).

Supplementary Table 4. iSNV calls in LASV LM032 rodent libraries

			Standard (12 total)		Hybrid selection (30 total)	
LASV segment	LASV genome position	Reference allele	Variant allele	Variant frequency (%)	Variant allele	Variant frequency (%)
L	1425	T	C	15	C	10.53
L	2889	C	T	23.44	T	25.02
L	5024	T	C	23.19	C	21.75
L	5579	A	G	25	G	30.52
L	6924	C	T	25.26	T	26.49
S	438	A	G	27.70	G	25.38
S	1097	C	T	23.39	T	26.24
S	1320	A	G	53.25	G	49.71
S	2179	C	G	8.10	G	5.85
S	2254	A	G	28.48	G	26.87
S	2843	T	C	25.28	C	22.41
S	2947	C	T	6.19	T	4.50
L	346	T			C	27.86
L	382	C			T	17.73
L	385	C			T	16.89
L	451	T			C	5.67
L	563	G			T	26.49
L	1436	T			C	5.38
L	2828	C			T	5.40
L	3357	A			G	6.39
L	5738	G			A	5.81
L	6203	G			A	5.10
L	7126	G			A	5.78
S	110	C			T	22.27
S	920	G			A	5.77
S	1289	A			G	16.86
S	1318	T			A	13.79
S	1432	G			A	13.10
S	1909	C			T	10.62
S	2899	T			C	5.60

All variants for standard and hybrid selection (> 4.5 minor allele frequency) were called using VarScan (see methods).
